# Palmitate-induced *Slc2a4*/GLUT4 downregulation in L6 muscle cells: evidence of inflammatory and endoplasmic reticulum stress involvement

**DOI:** 10.1186/s12944-018-0714-8

**Published:** 2018-04-02

**Authors:** Patrícia Ebersbach-Silva, Ana Cláudia Poletto, Aline David-Silva, Patrícia Monteiro Seraphim, Gabriel Forato Anhê, Marisa Passarelli, Daniela Tomie Furuya, Ubiratan Fabres Machado

**Affiliations:** 1 Department of Physiology and Biophysics, Institute of Biomedical Sciences, University of São Paulo, Av. Prof. Lineu Prestes 1524, São Paulo, 05508-900 Brazil; 2 Department of Physical Therapy, School of Science and Technology, Universidade Estadual Paulista, São Paulo, Brazil; 3 Department of Pharmacology, Faculty of Medical Sciences, State University of Campinas, Campinas, Brazil; 4 Laboratório de Lípides (LIM-10), Hospital das Clinicas HCFMUSP, Faculdade de Medicina, Universidade de Sao Paulo, Sao Paulo, SP Brazil

**Keywords:** Palmitate, GLUT4, ER stress, NFKB, L6 cells, Insulin resistance

## Abstract

**Background:**

Obesity is strongly associated to insulin resistance, inflammation, and elevated plasma free fatty acids, but the mechanisms behind this association are not fully comprehended. Evidences suggest that endoplasmic reticulum (ER) stress may play a role in this complex pathophysiology. The aim of the present study was to investigate the involvement of inflammation and ER stress in the modulation of glucose transporter GLUT4, encoded by *Slc2a4* gene, in L6 skeletal muscle cells.

**Methods:**

L6 cells were acutely (2 h) and chronically (6 and 12 h) exposed to palmitate, and the expression of several proteins involved in insulin resistance, ER stress and inflammation were analyzed.

**Results:**

Chronic and acute palmitate exposure significantly reduced GLUT4 protein (~ 39%, *P* < 0.01) and its mRNA (18%, *P* < 0.01) expression. Only acute palmitate treatment increased GRP78 (28%, *P* < 0.05), PERK (98%, *P* < 0.01), eIF-2A (35%, *P* < 0.01), IRE1a (60%, *P* < 0.05) and TRAF2 (23%, *P* < 0.05) protein content, and PERK phosphorylation (106%, *P* < 0.001), but did not elicit eIF-2A, IKK phosphorylation or increased XBP1 nuclear content. Additionally, acute and chronic palmitate increased NFKB p65 nuclear content (~ 30%, *P* < 0.05) and NFKB binding activity to *Slc2a4* gene promoter (~ 45%, *P* < 0.05).

**Conclusion:**

Different pathways are activated in acute and chronic palmitate induced-repression of *Slc2a4*/GLUT4 expression. This regulation involves activation of initial component of ER stress, such as the formation of a IRE1a-TRAF2-IKK complex, and converges to NFKB-induced repression of *Slc2a4*/GLUT4. These results link ER stress, inflammation and insulin resistance in L6 cells.

## Background

In the postprandial state, glucose uptake occurs mainly in skeletal muscle [1] through the insulin responsive glucose transporter GLUT4. Many reports have demonstrated that reduced GLUT4 expression in both skeletal muscle [2] and adipose tissue [3–5] is related to insulin resistance. Indeed, while the knockout of the *Slc2a4* gene, which encodes GLUT4 protein, results in insulin resistance [6], its overexpression ameliorates diabetes [7].

Insulin resistance states, such as diabetes and obesity, have been associated to low-grade systemic inflammation [3, 8, 9]. Recently, we have reported the participation of inflammation in insulin resistance by showing that the nuclear factor kappa B (NFKB) downregulates specifically the *Slc2a4* gene [10–13].

In addition to insulin resistance and inflammation, obesity is strongly related to elevated plasma free fatty acids (FFA), but the mechanisms behind this association are not fully comprehended. Recent evidences suggest that the endoplasmic reticulum (ER) stress may play a role in this complex pathophysiology.

Disturbed ER homeostasis results in unfolded and misfolded proteins, which can lead to cell dysfunction and death. In consequence of ER stress, an adaptive process named unfolded protein response (UPR) is triggered. UPR signaling is mainly mediated by ER transmembrane signal transducers, including the following: protein kinase R-like endoplasmic reticulum kinase (PERK), serine/threonine-protein kinase/endoribonuclease IRE1 (IRE1) and cyclic AMP-dependent transcription factor ATF-6 (ATF6). In non-stressed cells, 78 kDa glucose-regulated protein (GRP78), a central regulator of ER stress, binds and inactivates PERK, IRE1 and ATF6. In stressed cells, the accumulated unfolded proteins bind to GRP78, allowing the ER transmembrane proteins to be released from it and to become activated [14].

Palmitic acid (C16:0) is one of the most abundant saturated FFA in both human and rodent plasma [15]. It is able to induce peripheral insulin resistance, characterized by impaired glucose uptake by skeletal muscle [16]. As a matter of fact, it has been shown that palmitate is able to downregulate GLUT4 expression in skeletal muscle cells [17, 18] but little is known about the involved mechanisms.

The objective of the present study was to investigate the involvement of inflammation and ER stress in the modulation of *Slc2a4*/GLUT4 expression by acute and chronic palmitate exposure in L6 skeletal muscle cells.

## Methods

### Reagents

Rat L6 myoblasts (CRL 1458) were purchased from ATCC (APABCAM, Rio de Janeiro Brazil). Dulbecco’s-modified Eagle’s medium (DMEM) and fetal bovine serum (FBS) were from Vitrocell Embriolife (Campinas, SP, Brazil). Antibiotic (penicillin/streptomycin), bovine serum albumin (BSA), palmitate (P5585) and 3-(4,5-dimethylthiazol-2-yI)-2,5-diphenyltetrazolium bromide (MTT) were obtained from Sigma (St. Louis, MO, EUA). Trizol, Platinum SYBR Green qPCR SuperMix UDG and Taqman primers and probes were obtained from Life Technologies (Carlsbad, CA, USA), ImProm-II Reverse Transcriptase from Promega (Madison, WI, USA). Anti-GLUT4 (#07–1404) antibody was obtained from Millipore (Billerica, MA, USA), and anti-phospho-IKK-A/B (#2681) from Cell Signaling (Beverly, MA, USA). Anti phospho-PERK (Thr 981) (sc-32577), anti-PERK (H-300) (sc-13073), anti- eIF-2A (FL-315) (sc-11386), anti- IRE1a (H-190) (sc-20790), anti-PR15A (C-19) (sc-825), anti-DDIT-3 (F-168) (sc-575), anti-TRAF2 (C-20) (sc-876), and anti-NFKB p50 subunit (C-19) (sc-1190) antibodies were purchased from Santa Cruz Biotechnology (Santa Cruz, CA, USA). Anti-phospho-eIF-2A (Ser 51) (ab 32157), anti-XBP1 (ab 37152), anti-NFKB p65 subunit (ab 7970), and anti-GRP78 (ab 108615) antibodies were from Abcam (Cambridge, MA, USA), and horseradish peroxidase-linked immunoglobulin was from Amersham Biosciences (Buckinghamshire, UK).

### Cell culture and treatment

L6 cells were propagated and differentiated as previously reported [19]. Briefly, cells were propagated in growth medium [DMEM (25 mM glucose), 10% FBS and 1% (*v*/v) antibiotic] until reach 70% of confluence. Cells were then differentiated for 6 days in differentiation medium [DMEM (25 mM glucose), 2% FBS and 1% (*v*/v) antibiotic]. At seventh day, cells were incubated with FBS-free restriction/treatment medium [DMEM (5.5 mM glucose), 1% BSA and 1% (v/v) antibiotic] for 12 h before treatment. Palmitate was diluted in ethanol (vehicle) and added to a final concentration of 0.75 mM palmitate and 0.5% ethanol for 2, 6 and 12 h. In parallel, control plates were treated with a non-toxic final concentration of 0.5% ethanol [15, 20].

### Cell viability assay

L6 cells were plated in 12-well plate and treated with 0.5% ethanol alone for 12 h or with 0.75 mM palmitate/0.5% ethanol for 2, 6 or 12 h. Cell viability was assessed using MTT reagent according to the manufacturer’s recommendations with some modifications. Briefly, after the incubation with the tested compounds, the medium was changed to a medium with 10% MTT (5 mg/ml) and incubated in dark for 4 h at 37 °C. The medium was removed and replaced with a solvent solution (0.04 N HCl in absolute isopropanol), and the plates were placed on a plate shaker for 15 min to solubilize the formazan crystals derived from MTT reduction. The absorbance of the converted dye was measured at 570 nm with a background subtraction at 690 nm using a microplate reader (BioTek Microquant, BioTek Instruments, Winooski, VT, USA).

### Western blotting

Total membrane protein fraction for GLUT4 protein quantification was performed as previously reported [19]. For RE proteins (cytosolic proteins) extraction, L6 cells were removed from plates with PBS and centrifuged at 1500 g for 10 min at 4 °C. The resulting pellet was resuspended with sonication in ice-cold extraction buffer [1% SDS, 100 mM Tris (pH 7.4), 100 mM sodium pyrophosphate, 100 mM sodium fluoride, 10 mM EDTA, 10 mM sodium vanadate, 2 mM PMSF, and 0.1 mg/ml aprotinin], incubated at 96 °C for 10 min. and submitted to centrifugation at 15,000 g for 10 min at 4 °C. The resulting supernatants were frozen at − 20 °C or at − 80 °C. Nuclear proteins for Western blotting analysis were extracted from L6 cells as earlier described [11, 12]. Thirty micrograms of total protein were resolved by dodecyl sulfate polyacrylamide gel electrophoresis (10%T and 2.7%C for GLUT4, NFKB p50, NFKB p65, TRAF2, and XBP1; 12%T and 2.7%C for DDIT-3, phosho-eIF-2A, eIF-2A, IRE1a, phosho-PERK, and PERK; and, 8%T and 2.7%C for GRP78, phosho-IKK-A/B, PR15A), transferred to a nitrocellulose membrane, and immunoblotted for 12 h at 4 °C with specific primary antibody in the following concentrations: anti- DDIT-3 (F-168) (1:500, 1× TBS), anti-eIF-2A (FL-315) (1:500, 1× TBS), anti-phospho-eIF-2A (Ser 51) (1:2000, 1× TBS/3% BSA), anti-GLUT4 (1:3000 in 1× PBS/8% BSA), anti-GRP78 (1:500, 1× TBS/3% BSA), anti-phospho-IKK-A/B (1:500, 1× TBS/5% BSA), anti-IRE1a (H-190) (1:1000, 1× TBS/3% BSA), and anti-NFKB p50 (C-19) (1:500, 1× TBS/1% BSA), anti-NFKB p65 (1:500, 1× TBS/1% BSA), anti-PERK (H-300) (1:1000, 1× TBS/3% BSA), anti-phospho-PERK (Thr 981) (1:1000, 1× TBS/3% BSA), anti-PR15A (C-19) (1:1000, 1× TBS/3% BSA), anti-TRAF2 (C-20) (1:1000, 1× TBS/3% BSA), anti-XBP1 (1:1000, 1× TBS/3% BSA and antibodies. The membrane was then incubated with horseradish peroxidase-linked secondary antibody, and signal was detected by chemiluminescence. Blots were quantified by optical densitometry (ImageScanner III, GE Healthcare, Uppsala, Sweden). Because protocols of protein extraction of membrane (GLUT4), nuclear and cytosolic fractions are different, different approaches for protein-loaded normalization were undertaken. GLUT4 and nuclear protein content were normalized by analyzing post-transferring Coomassie Blue-stained gel [5, 21], and cytosolic protein content was normalized by ACTB content.

### Real time RT-PCR

One microgram of total RNA was extracted with Trizol. cDNA, obtained by reverse transcription, was then amplified with Taqman system. Commercially available Taqman primers for rat *Gapdh* (#Rn99999916_s1) was used for normalization. The primer sequences for rat *Slc2a4* were custom designed: 5 ′ GGCTGTGCCATCTTGATGAC-3′ (fw), 5′-CACGATGGACACATAACTCATGGA-3′ (rv) and FAM AACCCGCTCCAGCAGC MGB, as previously described [19].

### Eletrophoretic mobility shift assay (EMSA)

Nuclear proteins were extracted from L6 cells and subjected to EMSA as earlier described [12]. The sequence of the oligonucleotide specific for NFKB binding site in *Slc2a4* promoter (*Slc2a4*-NFKB) was 5`-GGGTTGG**GGGCGTGGCC**TTTTGG-3` [12, 19].

### Statistical analysis

Data are expressed as means ± S.E.M. One-way analysis of variance (ANOVA), with Student–Newman–Keuls as a post hoc test was used for analysis of comparison, after comparison of the variances by the Bartelett’s test.

## Results

### Evaluation of cell viability

**Fig. 1:**
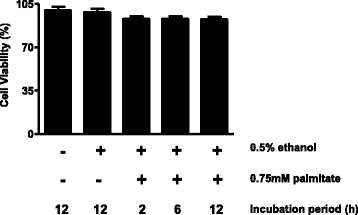
Evaluation of cell viability after exposure to ethanol (vehicle) and palmitate. L6 cells were treated with 0.5% ethanol alone for 12 h or 0.75 mM palmitate in 0.5% ethanol for 2, 6 or 12 h. Cell viability of L6 cells was evaluated by MTT assay. Values are means ± SEM of 12 to 20 samples; one-way ANOVA, Student–Newman–Keuls as post hoc test

The viability of L6 muscle cells incubated with 0.5% ethanol (vehicle) alone or with 0.75 mM palmitate was assessed with MTT assay. As shown in Fig. 1, treatment with palmitate as long as 12 h resulted in 92% of viable cells.

### Palmitate effects on *Slc2a4* gene expression in L6 myotubes

**Fig. 2:**
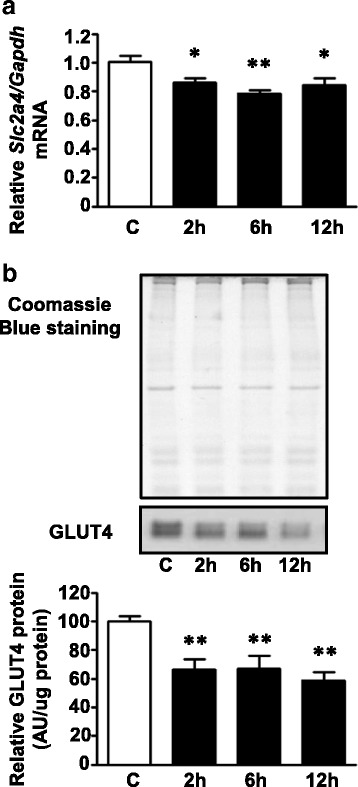
Palmitate reduces the expression of the *Slc2a4* gene in L6 myotubes. L6 myotubes were treated with 0.5% ethanol (vehicle) for 12 h (control sample, C) or 0.75 mM palmitate for 2, 6 or 12 h. **a** Relative *Slc2a4* mRNA normalized by *Gapdh* mRNA (RT-qPCR). **b** Relative GLUT4 protein normalized by total protein analysis with Coomassie Blue-stained gel (Western blotting). Values are means ± SEM of 6 to 10 samples; *P < 0.05 and **P < 0.01 versus C; one-way ANOVA, Student–Newman–Keuls as post hoc test

The effect of palmitate treatment on *Slc2a4* gene expression was investigated over a time course of 2, 6 and 12 h in L6 myotubes. We have previously demonstrated that 0.75 mM palmitate for 20 h reduces GLUT4 protein content and glucose uptake in L6 cells [18]. Here we showed that 0.75 mM palmitate reduced the *Slc2a4* gene transcript content (14, 22 and 16% after 2 h, 6 and 12 h, respectively, *P* < 0.05 and *P* < 0.01 versus C; Fig. 2a), and its encoded protein, GLUT4 (33% after 2 h and 6 h and 42% after 12 h, *P* < 0.01 versus C; Fig. 2b). As it can be observed, the repression effect of palmitate was greater in GLUT4 protein expression than in expression of its respective mRNA, suggesting the presence of a transcriptional and posttranscriptional regulation, such as UPR signaling. To test this hypothesis, the ER stress-mediated pathways were investigated.

### Palmitate effects on ER stress signaling pathways in L6 cells

**Fig. 3:**
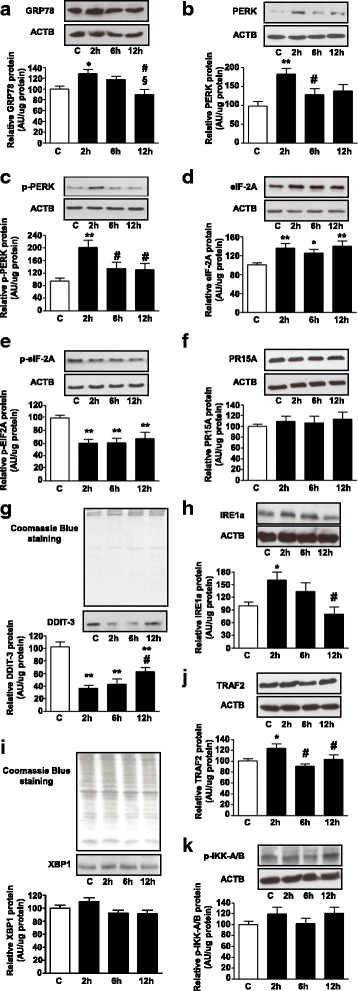
Palmitate effects on ER stress signaling in L6 cells. L6 myotubes were treated with 0.5% ethanol (vehicle) for 12 h (control sample, C) or 0.75 mM palmitate for 2, 6 or 12 h. Relative cytosolic (**a**-**f**, **h**, **j**, and **k**) or nuclear (**g** and **i**) protein content. Protein content was normalized by ACTB (**a**-**f**, **h**, **j**, and **k**), or by total protein analysis with Coomassie Blue-stained gel (G and I). Values are means ± SEM of 7 to 8 (**a**), 6 to 7 (**b**), 8 to 12 (**c**), 10 to 11 (**d**), 7 to 8 (**e**), 4 to 6 (**f**), 8 to 12 (**g**), 6 to 9 (**h**), 9 to 10 (**i**), 5 to 7 (**j**), and 8 to 9 (**k**) samples; *P < 0.05 and **P < 0.01 versus C; #*P* < 0.05 versus 2 h, §*P* < 0.05 versus 6 h; one-way ANOVA, Student–Newman–Keuls as post hoc test

L6 muscle cells treated with palmitate for 2 h showed an increase in GRP78 protein content (28%, *P* < 0.05 versus C, Fig. 3a), which was subsequently dissipated. Thus, two branches of ER stress pathway were investigated including PERK and IRE1.

PERK activation is the most immediate response to ER stress. In fact, as a consequence of increased GRP78 protein content (Fig. 3a), L6 cells showed enhanced PERK total content (98%, *P* < 0.01 versus C, Fig. 3b) and phosphorylation (106%, *P* < 0.001 versus C, Fig. 3c) after 2 h-palmitate treatment. Further, PERK activation is usually followed by phosphorylation of eukaryotic translation initiation factor 2 subunit 1 (eIF-2A); interestingly, while palmitate increased eIF-2A protein content (35, 25 and 39% after 2 h, 6 and 12 h, respectively; *P <* 0.05 and *P* < 0.01, versus C; Fig. 3d), it decreased eIF-2A phosphorylation (41, 40 and, 33% after 2, 6 and 12 h, respectively; *P* < 0.01 versus C; Fig. 3e).

In order to further investigate the discrepancy between the eIF-2A content and phosphorylation, a phosphatase that dephosphorylates eIF-2A, PR15A (protein phosphatase 1 regulatory subunit 15A), also known as growth arrest and DNA damage-inducible protein 34 (GADD34) was analyzed. Surprisingly, palmitate did not exert any effect in PR15A content in L6 cells (Fig. 3f).

The activation of PERK regulates numerous genes involved in apoptosis such as DDIT-3 (DNA damage-inducible transcript 3 protein), also known as growth arrest and DNA damage-inducible protein (GADD153) [22]; thus, the effect of palmitate on the expression of this protein was investigated. Interestingly, palmitate reduced the nuclear content of DDIT-3 3 (66, 60 and 40%, after 2, 6 and 12 h, respectively; *P* < 0.01 versus C; Fig. 3g) indicating that PERK signaling was not activated downstream.

Next, another pathway of UPR signaling was investigated. In parallel to the increased GRP78 protein content after 2 h-palmitate incubation, the content of protein kinase IRE1a also increased (60%, *P* < 0.05 versus C, Fig. 3h). The activation of IRE1 results in spliced isoform of XBP1, which positively regulates genes involved in UPR signaling [23]. The present work used an anti-XBP1 antibody that recognizes both spliced and unspliced isoform of XBP1. Here, acute palmitate slightly, but not significantly, increased XBP1 protein content (Fig. 3i).

Alternatively, ER stress can also trigger IRE1a to interact with the kinase adapter tumor necrosis factor receptor-associated factor 2 (TRAF2) and form a complex with inhibitor of nuclear factor kappa-B kinase (IKK) promoting activation of NFKB, independently of IKK activation [24]. Here, it was found that TRAF2 increased (23%, *P* < 0.05 versus C, Fig. 3j) in L6 myotubes after 2 h-palmitate incubation, albeit IKK phosphorylation was not modified (Fig. 3k).

### Participation of NFKB transcription factor in the *Slc2a4* gene downregulation by palmitate in L6 muscle cells

Finally, the participation of NFKB in the modulation of *Slc2a4* gene by palmitate was investigated.

**Fig. 4:**
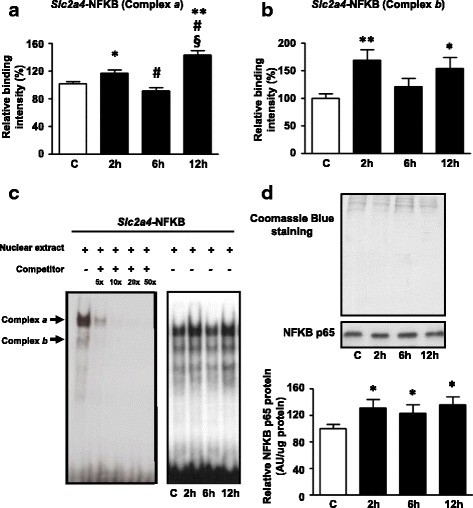
NFKB participates in *Slc2a4* gene downregulation by palmitate in L6 cells. L6 myotubes were treated with 0.5% ethanol (vehicle) for 12 h (control sample, C) or 0.75 mM palmitate for 2, 6 or 12 h. **a** and **b** Relative nuclear protein content of complexes *a* and *b* (*Slc2a4*-NFKB); **c** EMSA competition assay (left panel) and representative image of EMSA assay showing complexes *a* and *b* (right panel); **d** Relative NFKB p65 nuclear protein content normalized by total protein analysis with Coomassie Blue-stained gel. Values are means ± SEM of 10 (**a**), 9 to 10 (**b**), and 5 to 10 (**d**) samples; *P < 0.05 and **P < 0.01 versus C; #*P* < 0.05 versus 2 h, §*P* < 0.05 versus 6 h; &*P* < 0.05 versus 12 h, one-way ANOVA, Student–Newman–Keuls as post hoc test

Recently, we have reported that NFKB downregulates the *Slc2a4* gene; in L6 muscle cells, the binding of NFKB dimers to a double-stranded oligonucleotide containing a NFKB site sequence inside the *Slc2a4* promoter results in two complexes named *a* and *b* [12]. According to our previous report [12], we confirmed here the oligonucleotide binding specificity by competition assay with unlabeled probe (Fig. 4c). Additionally, we have previously confirmed the specific binding of NFKB p50 and p65 subunits in the protein/DNA binding complex in L6 cells. As shown in Fig. 4a and b, treatment with palmitate increased NFKB binding activity after exposure of 2 h (16% for complex *a*, *P* < 0.05 versus C; and, 68% for complex *b*; *P* < 0.01 versus C), and after 12 h (42% for complex *a*; *P* < 0.01 versus C; and, 53% for complex *b*; *P* < 0.05 versus C). Additionally, palmitate increased the nuclear content of NFKB p65 in L6 cells (30, 23 and 32% after 2, 6 and 12 h, respectively; *P* < 0.05 versus C, Fig. 4d).

## Discussion

Palmitate is known to induce insulin resistance by imparing glucose uptake [16] and reducing GLUT4 expression [17, 18] in skeletal muscle; nonetheless, little is known about the involved mechanisms.

Here we reported that palmitate reduced GLUT4 expression in L6 muscle cells; still, the correlation of mRNA and protein content was not consistent. Since post-transcriptional modulation has been reported to cause discrepancies between GLUT4 protein and its transcript abundancies [25], it was hypothesized that ER stress could participate in the modulation of the *Slc2a4* gene. Actually, it has been reported that activation of UPR signaling represses the *Slc2a4* gene in 3 T3-L1 adipocytes [26]. Also, it has been reported that palmitate activates UPR system in hepatocytes [27], pancreatic beta-cells [28], and C2C12 mouse muscle cell [29]. Up to date, only one report has shown that palmitate (1 mM, 24-h treatment) induces ER stress in L6 cells [30]. The present findings showed that lower concentration of palmitate (0.75 mM) acutely (2 h) and transiently induced the expression and activation of proteins related to the initial steps of UPR signaling in L6 myotubes. In fact, palmitate has a weaker effect on UPR compared to tunicamycin, a chemical ER stress inducer in C2C12 myotube [31]. Here, we showed that acute palmitate was able to increase the expression of GRP78 protein, a chaperone positively regulated in stress conditions. When unfolded proteins bind to GRP78, transmembrane proteins from ER such as IRE, PERK and ATF6 are released from GRP78 binding and become activated [14]. Here we investigated two branches of UPR pathway, PERK and eIF-2A.

Acute palmitate exposure resulted in increased expression of PERK and eIF-2A; and surprisingly, while PERK activation was increased, eIF-2A phosphorylation was reduced. Some phosphatases such as PR15A can dephosphorylate eIF-2A, attenuating response to stress and promoting recovery of protein synthesis [32]. In our study, since no alteration on PR15A was observed by palmitate treatment, it is suggested that other factors may be involved in the observed intense reduction of eIF-2A phosphorylation. Besides, the activation of PERK regulates numerous genes involved in apoptosis such as DDIT-3 [22]. Here, we found a decrease in DDIT-3 nuclear content pointing out that acute palmitate, at the present concentration, does not result in apoptosis in L6 myotubes, as confirmed by the unchanged cellular viability observed up to 12 h of culture.

We also demonstrated that acute palmitate even though was able to increase IRE1a expression, it did not result in its activation since phosphorylation of IRE1a and XBP1 nuclear content was not altered. However, our results strongly suggest that IRE1a can contribute to NFKB activation after acute palmitate exposure. It has been reported that basal IKK activity is sufficient to activate NFKB by IRE1a, and this occurs by physical interaction among IRE1a, TRAF2 and IKK [24]. The formation of this complex is transient and can occur in basal state, even if there is no activation of UPR signaling [24]. Albeit increased IKK phosphorylation was not observed after acute palmitate treatment, increased IRE1a and TRAF2 protein expression, besides NFKB p65 activation were observed suggesting that the formation of the complex IRE1a-TRAF2-IKK may activate NFKB in L6 cells after 2 h-palmitate exposure. Besides this non-classical NFKB activation, other IKK phosphorylation-independent pathways have been described. One example of this could be the direct phosphorylation of NFKB p65 by protein kinases such as PKC and PKA, independently of IKK [33–35]. Another example could be the activation of NFKB in nuclear factor-kappa-B inhibitor (IKB)-depleted cells [36].

Recently, it has been demonstrated the rat/mouse *Slc2a4* gene promoter has a functional kB site to which NFKB binds and downregulates its transcription [12]. Additionally, many studies have reported that reduced GLUT4 expression occurs in insulin resistant states [2–5]. Thus, NFKB signaling is a prominent molecular signaling that links inflammation to insulin resistance. The present data point out that 2 h- and 12 h-treatment with palmitate increases the nuclear content of NFKB p65 subunit and NFKB binding specifically to the *Slc2a4* promoter, and consequently reduces *Slc2a*/GLUT4 expression. Consistent with these results, we have previously demonstrated that the same concentration of palmitate for 20 h reduces GLUT4 expression and glucose uptake; moreover, it induces tumor necrosis factor gene expression and NFKB accumulation in the nucleus in L6 cells [18]. Our data suggest that while acute palmitate treatment induces NFKB by the formation of IRE1a-TRAF2-IKK complex, chronic treatment effect should occur by some other non-classical pathway. In addition, treatment with palmitate for 6 h also reduced the expression of *Slc2a4*/GLUT4 in L6 cells, but did not increase NFKB binding in *Slc2a4* gene promoter. Of note, a different pathway should be involved.

**Fig. 5:**
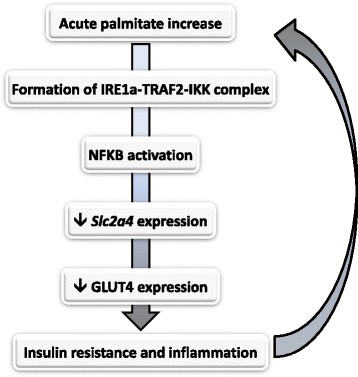
Participation of ER stress in palmitate-induced inflammation and insulin resistance in skeletal muscle cells. Acute palmitate treatment induces the physical interaction among IREa, TRAF2 and IKK, resulting in the formation of IREa-TRAF2-IKK complex. This complex activates NFKB nuclear factor, which translocates to the nucleus and binds to the *Slc2a4* gene promoter, downregulating it and leading to a decreased expression of GLUT4 protein, and, consequently to insulin resistance. In vivo, this mechanism could be triggered by high-fat meals, favoring the establishment or the worsening of muscle metabolic disorders related to insulin resistance

In summary, the data reveal that short-term exposition of muscle cells to palmitate can repress *Slc2a4*/GLUT4 expression, in a ER- and inflammatory-stress mediated way; thus, contributing to induce insulin resistance (Fig. 5). Considering that, we highlight that the increased consumption of fats, as observed in Western diet and processed foods, may contribute to acute and intermittent increases in circulant FFA levels, favoring the establishment or worsening of muscle metabolic disorders related to inflammation, insulin resistance and diabetes onset.

## Conclusion

Palmitate reduces *Slc2a4*/GLUT4 expression in L6 muscle cells. That is related to formation of a IRE1a-TRAF2-IKK complex, activation of NFKB and repression of *Slc2a4* gene transcription. These findings indicate that ER stress may connect palmitate-induced inflammation and insulin resistance in L6 muscle cells.

## Abbreviations

BSA: Bovine serum albumin
DMEM: Dulbecco’s-modified Eagle’s medium
EMSA: Electrophoretic mobility shift assay
ER: Endoplasmic reticulum
FBS: Fetal bovine serum
FFA: Free fatty acids
MTT: 3-(4,5-dimethylthiazol-2-yI)-2,5-diphenyltetrazolium bromide)
UPR: Unfolded protein response
ᅟ: Acronyms used for genes and proteins were based on the Rat Genome Database (RGD) and the Protein Knowledgebase (UniProtKB) respectively

## Genes

*Actb*: Actin beta
*Gapdh*: Glyceraldehyde-3-phosphate dehydrogenase
*Slc2a4*: Solute carrier family 2 member 4

## Proteins

ACTB: Actin cytoplasmic 1 (former beta-actin)
ATF6: Cyclic AMP-dependent transcription factor (indiscriminate subunit)
DDIT-3: DNA damage-inducible transcript 3 protein (former growth arrest and DNA damage-inducible protein 153, GADD153)
eIF-2A: Eukaryotic translation initiation factor 2 subunit 1
GLUT4: Glucose transporter 4; GRP78, 78 kDa glucose-regulated protein
IKB: Nuclear factor-kappa-B inhibitor (former IκB)
IKK-A/B: Inhibitor of nuclear factor kappa-B kinase subunits alpha/beta (former IKKα/β)
NFKB: Nuclear factor NF-kappa-B (indiscriminate isoform)
PR15A: Protein phosphatase 1 regulatory subunit 15A (former growth arrest and DNA damage-inducible protein 34, GADD34)
IRE1: Serine/threonine-protein kinase/endoribonuclease IRE1
PERK: Protein kinase R-like endoplasmic reticulum kinase
PKA: cAMP-dependent protein kinase catalytic subunit
PKC: Protein kinase C
TRAF2: Tumor necrosis factor receptor-associated factor 2
XBP1: X Box Protein-1
